# The effect of electron dose on positive polymethyl methacrylate resist for nanolithography of gold bowtie nanoantennas

**DOI:** 10.1016/j.heliyon.2022.e09475

**Published:** 2022-05-18

**Authors:** Caroline Campbell, Abigail Casey, Gregory Triplett

**Affiliations:** a Mechanical and Nuclear Engineering, Virginia Commonwealth University, Richmond, VA, 23220, USA; b Chemical and Life Sciences Engineering, Virginia Commonwealth University, Richmond, VA, 23220, USA; c Electrical and Computer Engineering, Virginia Commonwealth University, Richmond, VA, 23220, USA

**Keywords:** Bowtie nanoantennas, Surface Enhanced Raman Spectroscopy (SERS), Fabrication, Electron beam lithography, Nanopatterning, Exposure, Dose, Monte Carlo simulation, Polymethyl methacrylate (PMMA)

## Abstract

Plasmonic structures, such as bowtie nanoantennas, may be used in Surface Enhanced Raman Spectroscopy (SERS). Nanoantennas can be employed to amplify the biomolecular and chemical reactions, which is useful for biomedical applications. The electric field created by nanoantennas are optimized when the resonant wavelength of the probed laser light closely matches the resonant wavelength of the plasmonic structure. In this work, we fabricated several bowtie nanoantennas with varying geometric spacing for use with a 532 nm wavelength laser line in Raman Spectroscopy. The fabrication utilized nanolithography by electron beam lithography on a Raith Voyager, development, deposition, and metal lift-off. This study explored a specific bowtie nanoantenna geometry of 270 nm equilateral sides triangle pairs with 3 varying gap sizes, 50 nm, 20 nm, and 10 nm, and the effect of varying electron beam doses on the final structure of the nanoantenna. The results presented here, will show that the working dose factor range is 6.5–10.3 (650–10,300 μC/cm^2^) for 120 nm thick polymethyl methacrylate (PMMA), and with a 44.78% increase in dose, the footprint area increases between 5.9% and 10.7%.

## 1 Introduction

The key to early disease detection is understanding signal transduction. Signal transduction, characteristic of all living cells, is a process where external molecular signals trigger internal cellular response. It affects memory function, muscles, and metabolism. Signal transduction is the communication process on the macro-level between cells. There are several main steps: the cell wall receives an external signal, the signal is translated and transferred as a message internally, and a response is triggered. That triggered response performs a specific function or resends the message to another cell where the process restarts. This is a chain reaction and each event begets a response. Diseases like cancer [1, 2, 3, 4], Alzheimer's [1, 2, 4, 5], Parkinson's [1, 2, 4], diabetes [1, 2, 6], and Huntington's [1, 2] can all be attributed to misfolding proteins which is essentially a failed signal transduction [1]. However, precisely monitoring signal transduction mechanism remains difficult [7]. In order to study these processes as they occur, it is critical to leverage and integrate technologies that provide a window of opportunity to record events without inducing any artifacts or changes to the cell's environment [8, 9]. A pathway for monitoring these events has been identified, and they include integration of proven technologies that enable the study of biochemical reactions, such as protein phosphorylation [10].

In order to monitor reactions in real time, signal enhancement is necessary to raise the detected Raman signal of the biochemical reaction (signal transduction) above the Raman background noise. Signal enhancement through surface enhanced Raman scattering (SERS) is a phenomenon and is the exchange between photons and molecular vibrations by the electromagnetic fields [11, 12]. This technique is more sensitive compared to regular non-enhanced Raman signal because it is employed at an excitation wavelength similar to the resonance of the probed molecule [12]. This signal enhancement through SERS can be achieved by the localized surface plasmon resonance, available through the metal nanoantennas [12]. The field enhancement of the nanoantenna is due to the metallic properties of the conducting layer as well as the geometry of the nanoantenna in terms of shape, size, gap size, and pair spacing.

**Figure 1:**
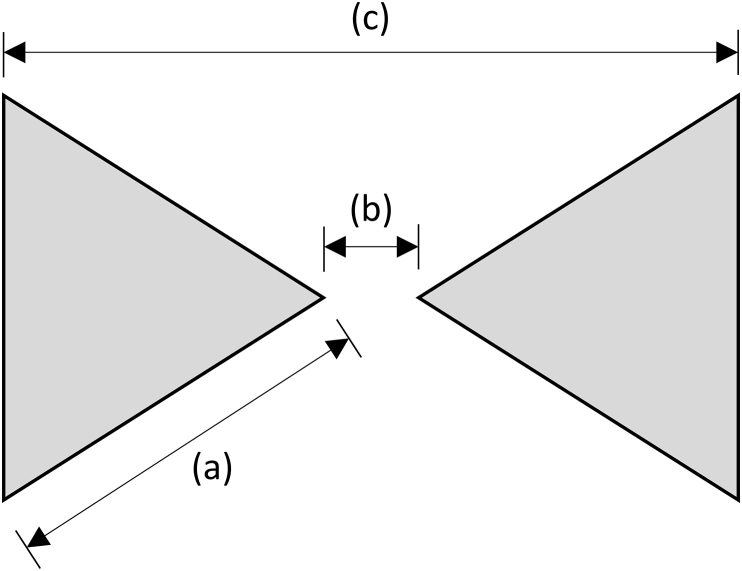
A drawing detailing the (a) side length, (b) tip-to-tip gap, and (c) total width for the bowtie nanoantennas.

Nanoantennas come in a variety of shapes. Specifically, the popularity of employing a nanoantenna with the bowtie geometry lies in its ability to enhance an electromagnetic field in a controlled, nanoscale space [13]. Bowtie nanoantennas are a popular choice as they can exhibit a higher electric field as compared to other geometries like rods and circles [14, 15]. The electric field at the surface of the nanoantenna is proportional in size to the radius of curvature of the edge of the structure [16, 17]. Therefore, sharp pointed structures offer greater field intensity than gently curved structures. A simple schematic of a bowtie nanoantenna is shown below in Figure 1. Nanoantennas can be fabricated using electron beam lithography and their geometry is limited by the resolution of the instrument [18]. This higher electric field is due to the plasmons in the surface layer of the conductive material moving from the closest location to another, in the tip-to-tip gap. Locally isolated electric fields can be used to enhance Raman spectroscopy. These locally isolated fields are produced through devices called nanoantennas, which are nanoscopic structures of a predetermined shape and size [19]. These electric fields are formed as a result of the excitation of localized surface plasmons and occur at the interface of conductive and dielectric materials [20]. [21]. Noble materials are used in nanoantennas because there is an optical response in the visible wavelength from the localized surface plasmon resonance phenomenon [22, 23, 24] as well as increased sample life. The signal enhancement ranges from 2 to 8 orders of magnitude depending upon the geometry of the paired device, the excitation wavelength, and the properties of the probed molecule [12]. Nanoantennas have a variety of applications, but one of the most prevalent is for Surface Enhanced Raman spectroscopy (SERS) [25]. SERS is the coupling of Raman spectroscopy and electric fields created by nanostructures in order to detect phase transitions and chemical reactions [19]. One of the main applications of SERS is in the biomedical field from fundamental bench top studies through clinical diagnostic work [26]. The penetration depth of light in tissue has increased the interest for SERS in biomedical optics [27]. Nanostructures for SERS have been used to detect DNA and other small molecules.

The nanoantennas in this work compare the exposure doses between three different geometric spacing of bowtie nanoantennas in order to find a dose working range as part of a metal lift off fabrication process. This metal lift off process employed in this work is a common technique to produce standalone nanostructures that are fabricated for a variety of applications, such as enhancing Raman spectroscopy or on-chip sampling of optical fields [28]. The geometries discussed in this work are equilateral triangles with a side length of 270 nm with a 50 nm tip-to-tip gap, a 20 nm tip-to-tip gap, and a 10 nm tip-to-tip gap. They are fabricated via a five-step process: applying resist, exposure, development, deposition, and metal removal. Resist is a polymer mask used to shield the substrate from metal deposition. Exposure is the step for where the polymer mask is bombarded with an electron beam and a portion of the polymer chains break into smaller chains. Exposing the polymer mask is a critical part of the nanoantenna fabrication process and an overexposure can lead to an expansion in the overall size of the nanoantenna, negatively affecting the performance in producing an electric field. Similarly, underexposing the mask could lead to underdosing, making a smaller than desired footprint which would increase the tip-to-tip gap. Proximity Effect Correction (PEC) software could be used to minimize the exposure due to backscattering electrons [29]. This work does not use PEC software in order to further the understanding of the effect of dose on positive PMMA resists.

In the development step, the smaller polymer chains are preferentially dissolved [30, 31]. Specifically, the smaller molecular chains are dissolved in a developer, a type of solvent, while the larger chains remain; this dissolution creates a cavity in the polymer mask in the shape of the footprint of the desired nanoantenna geometry [30, 31]. That cavity is subsequently filled with the deposited materials.

**Figure 2:**
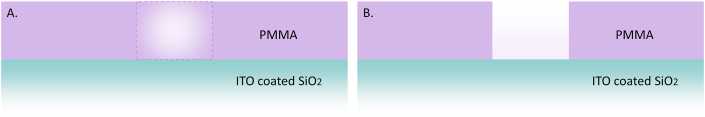
A cross section of PMMA on the ITO/SiO_2_ substrate with the (A) active exposed area during exposure, but before development and (B) after exposure, after development.

Exposure and development are shown in Figure 2. In order to understand the criticality of the electron beam interactions with the mask and substrate layers, CASINO, a Monte Carlo simulation software, was used to aid in the understanding how the electron beam interacts with material boundaries and different compositions [9].

Deposition is the process of applying a thin film to the substrate. This work will utilize electron beam evaporation deposition (EBED). After deposition of the desired materials has completed, the excess material is removed by dissolving the remaining polymer mask.

## 2 Methodologies

The work was fabricated in a class 1000 cleanroom environment at the Virginia Microelectronics Center. Bowtie nanoantennas are fabricated on indium tin oxide (ITO) coated glass substrates. After cleaning the substrate with acetone and isopropanol (IPA), the substrate was coated with polymethyl methacrylate (PMMA) (purchased from Kayaku, previously known as Microchem), a positive resist used for electron beam lithography. For these samples, the resist is 120 nm thick and is composed of 4 layers. Two layers of 495,000 and two layers of 950,000 molecular weight PMMA A2, which is 2% solids dissolved in anisole. The ratio of PMMA A2 molecular weights was 1:1 495,000:950,000 by volume. The ITO/glass substrate was spin coated with the PMMA resist at a stepped program: 5s at 500 rpm, then 45 s at 3500 rpm, then 5s at 300 rpm. The ITO/glass substrate was baked after coating with each resist layer for 2 min at 115 °C.

Lithography was done using a Raith Voyager with an excitation voltage of 50 kV and a beam current of 309.7 pA, which has a beam current deviation less than ± 0.2%/hour. The Voyager has automatic system calibrations and is designed for high speed patterning. The Raith Voyager uses a 50 MHz, 18 bit, pattern generator and patterns are created in the Raith Nanosuite with GDSII CAD Editing software. The Voyager has a 1 cm^2^/hour writing speed and this work uses the 500 μm writefield and has a beam stability deviation less than 120 nm/8 h.

After lithography, all samples were submerged in cold 4°Cmethyl isobutyl ketone (MIBK) and IPA at a 1:3 MIBK:IPA ratio, a developer, and rinsed with IPA. Cold development was used in order to increase feature contrast and decrease sensitivity [32]. In previous work from our group it was determined that the optimal development range was between 30 and 40 s [18]. These samples were developed for 36 s [18]. After development, the sample was placed in the vacuum chamber of the electron beam evaporation deposition system to reach a final high vacuum, 2.0 × 10^−8^ Torr, before deposition. Chromium (5 nm) was deposited at 0.2 Å/s as an adhesion layer followed by the gold conductive layer deposited at 0.2 Å/s ramping down to 0.1 Å/s six minutes before target film thickness was reached (30 nm). After deposition, the samples were left to soak in Remover PG (Kayaku) for 2 h at 70 °C with stirring at 70 rpm as a part of the metal lift off process. Remover PG was chosen as a solvent for the metal lift-off phase as recommended by the manufacturer. Any remaining metal deposition was allowed to dissolve in acetone for five minutes before a final wash with IPA to clear remaining traces of acetone. The nanoantennas are 270 nm equilateral triangles with varying tip-to-tip gap sizes at 50 nm, 20 nm, and 10 nm. The pattern created was a dose matrix to analyze the possible geometry and gap combinations. The dose matrix is seen in the figure below and consists of 20 different dose factor arrays for three different gap sizes.

**Figure 3:**
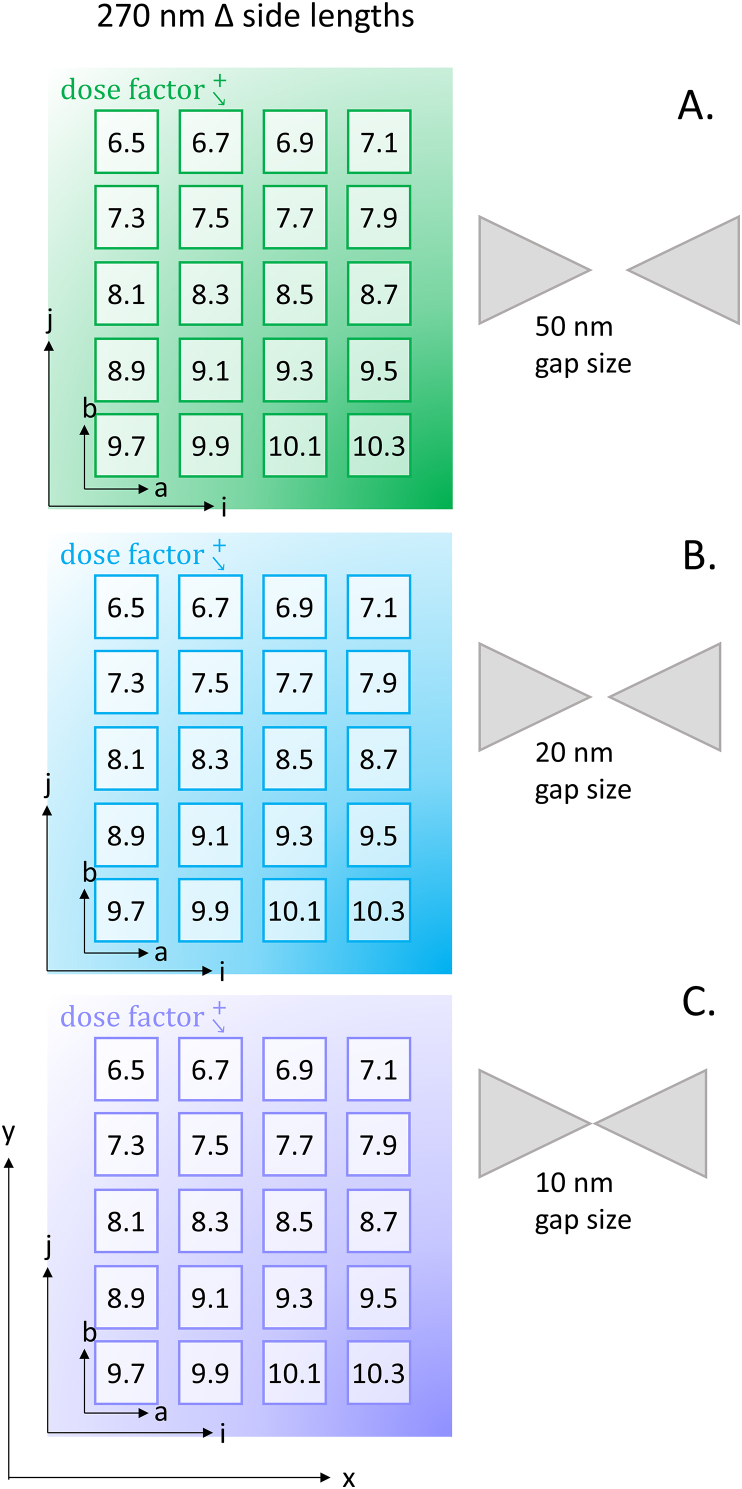
The Dose factor Map is an illustration showing twenty dose factors, ranging from 6.5 to 10.3, for three separate gap sizes of 50, 20, and 10 nm in the A., B. and C. schematic, respectively. The (a,b) axis belongs to each dose array and is denoted with a number within the 6.5–10.3 range, where there are 7,000 bowtie nanoantennas internally, and is approximately 150 μm by 200 μm. The (i,j) axis belongs to each geometrical spacing, illustrated as either green (A.), blue (B.), or purple (C.), and each geometrical spacing is approximately 1,100 μm by 1,600 μm (1.1 mm, 1.6mm). The (x,y) axis belongs to the entire pattern. The entire pattern is approximately 5.8 mm by 1.6 mm.

The dose is energy bombardment per area and typical units are μC/cm^2^. The long molecular chains of the polymer mask undergo molecular scissioning and break apart into smaller polymer chains when energy is applied [18, 32, 33]. The figure above, Figure 2, demonstrates a simplified cross-section of a polymer mask on a substrate with an active area of molecular scissioning. The Dose factor is a multiplier applied to a constant base dose. The base dose being 100 μC/cm^2^. The multiplication of those two amounts results in the final dose for the designated pattern. A visual representation of the dose factors is shown below in Figure 3. Each sample contained twenty separate dose arrays, ranging from 650 – 10,300 μC/cm^2^. In each dose array, there is a nanoantenna geometry of a triangle side length of 270 nm, and a gap distance of 50, 20, or 10 nm. These dose factor arrays had an internal array of the aforementioned geometry containing 7,000 bowtie nanoantenna pairs (or 14,000 standalone structures) for the 270 nm side length triangle. There are a total of 84,000 nanoantenna pairs, or 168,000 individual structures in the four dose factors studied here. Evaluated nanoantenna pairs were chosen at random due to the total number of nanoantenna pairs available. Previous simulations show that this geometry will have a relatively high electric field [34].

Predictability is a crucial element in mass fabrication. Polymer mask exposure is a key element in fabricating the structure as designed to optimize local electric field intensity. Underexposure could lead to a smaller than desired footprint which could increase the gap size, and thereby lower the electric field [34]. Overexposure could create a larger cavity which may cause the tips of the triangles to fuse, or create sloped sidewalls. For this reason, it is crucial to determine the working range for a specific polymer mask and nanoantenna fabrication recipe. The range that this work has found to be acceptable is 650–10,300 μC/cm^2^ for a PMMA layer 120 nm thick.

Monte Carlo simulations were performed using Casino at 50 kV with varying electron counts to estimate the achievable aspect ratio using PMMA. Monte Carlo is a numerical solution simulation solver approach that analyzes and projects results from a variety of parameters and boundary conditions [35, 36, 37, 38, 39]. The approach is based on a single photon scattering from an interaction with media [35].

Monte Carlo simulations are the repeated and random sampling of data in order to produce numeric results. Monte Carlo simulations offer the following advantages: any scattering parameter matrix can be used, the calculation time is relatively small compared to other simulation engines, additional detectors can be added without noticeably increasing calculation time, the ease of use as it relates to material parameters, and allows the ability to model with complex geometry and optical properties [35]. Monte Carlo simulations are used to investigate polarized light scattering and propagation in literature [40, 41, 42, 43, 44, 45, 46, 47, 48, 49, 50, 51, 52]. Beam size was estimated to be a circular 5nm for the Raith Voyager, and CASINO assumes a Gaussian beam with the user specified beam diameter being 99.9% of the beam [53]. These CASINO simulations can also show the penetration depth of the electrons, the energy of the back scattered electrons and their trajectories, the radius of the backscattered electrons, as well as the quantity of the backscattered electrons through the backscattering electron coefficient [53]. These simulations illustrate the potential for beam spread and backscattering electrons that could overexpose the polymer mask. This overexposure due to back scattering is also referred to as the proximity effect and should be taken into account when trying to determine the appropriate parameters for a pattern [54, 55].

**Figure 4:**
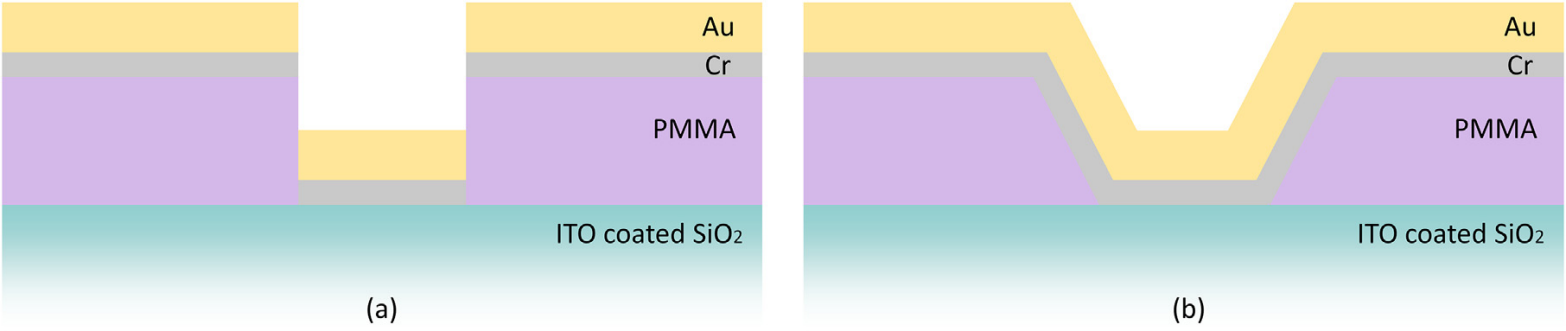
A simplified cross section of deposition with (a) ideal exposure and (b) exposure impacted by severe electron backscattering.

The schematics below in Figure 4 describe the ideal development and deposition case and a failed case, an overexposure possibly due to excess backscattering electrons. These are additional reasons for the criticality of appropriate dose and the importance in identifying a working range. Ideally, the cavity created by the previous steps would allow for the deposited thin metal films to avoid connection with each other. If the dose was too high, then the inflated footprint could have sloped sidewalls, leading to connections between metal thin films. When the polymer mask is dissolved, the connected metal would rip from the substrate and cause a failed nanoantenna. The edges could be sheared, jagged, or the nanoantenna may not be present in its entirety.

## 3 Results and discussion

Understanding backscattered electrons and the proximity effect through CASINO may help guide nanoantenna fabrication efforts to compensate for overexposure [53, 56]. The figure below shows the electron beam penetrating the PMMA, ITO, and glass (SiO_2_) layers. Previous work from our lab illustrated that higher accelerating voltage for the electron beam resulted in better aspect ratios for nanolithography [18].

**Figure 5:**
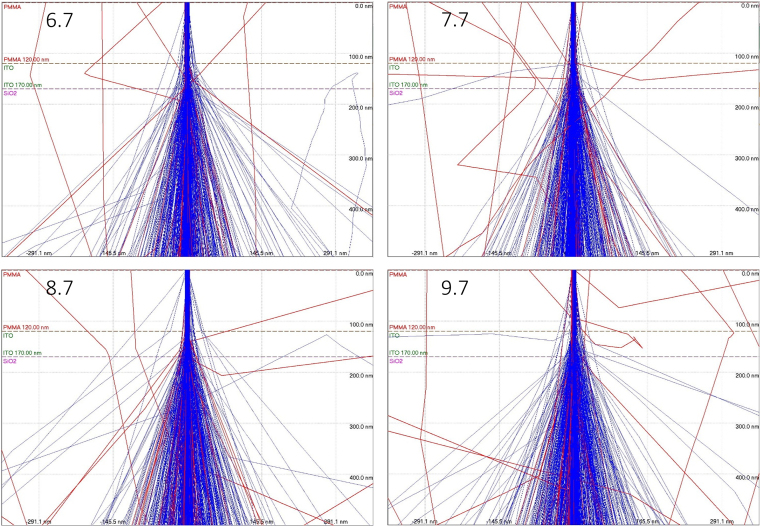
Images taken from CASINO showing the interaction of the 6.7 (821), 7.7 (944), 8.7 (1066), and 9.7 (1189) Dose factor (electrons) interacting with PMMA, ITO, and SiO_2_ with absorbed or transmitted (blue) and backscattered (red) electrons.

The Monte Carlo simulations, shown in Figure 5, at 6.7, 7.7, 8.7, and 9.7 dose factors received a dose of 670 (821), 770 (944), 870 (1066), and 970 (1189) μC/cm^2^ (electrons). In Figure 5, the colors of the lines in the Monte Carlo simulation images correspond to electrons transmitted or absorbed (blue) or backscattered electrons (red). The same parameters were used for all simulations, with the only difference being the number of electrons used to correspond with the electron dose delivered. The red back scattered lines demonstrate how the PMMA mask layer can be additionally exposed through reentry and exit of the backscattered electrons. These Monte Carlo simulations output the backscattering coefficient, which corresponds to the ratio of backscattered electrons as compared to the total number of electrons used [56]. As the dose used for the pattern increases, so does the total electron count and the total number of backscattered electrons. The backscattered electron coefficient (BEC) is calculated using Eq. (1).

(1) $$BEC=\frac{Backscattered\;Electron\;Count}{Electron\;Count\;in\;Beam}$$

An excess of backscattered electrons could over expose the polymer mask. Table 1 details the dose, the total number of electrons used, and the total number of backscattered electrons.

**Table 1:** The Dose, electron count in beam, and CASINO backscattered electron coefficient with the calculated backscattered electron count.

Dose (μC/cm^2^)	Electron Count in Beam	Backscattered Electron Coefficient	Backscattered Electron Count
970	1189	0.07016714286	83.42873286
870	1066	0.07290285714	77.71444571
770	944	0.07400128571	69.85721371
670	821	0.06786142857	55.71423286

**Figure 6:**
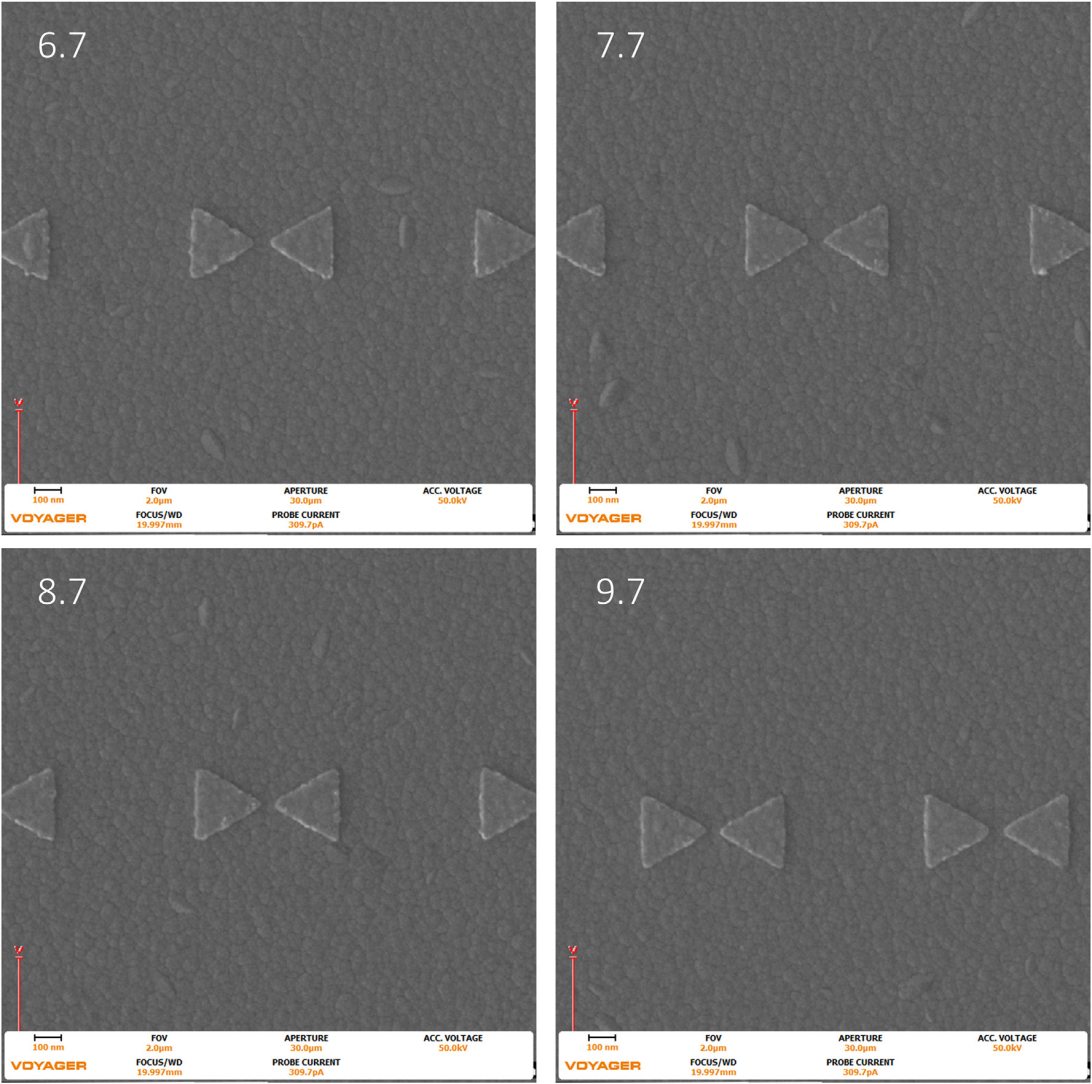
Images were taken with the Raith Voyager showing a 2 μm field of view (FOV) 6.7 (670), 7.7 (770), 8.7 (870), and 9.7 (970) Dose factor (μC/cm^2^) set of gold bowtie nanoantennas with a programmed geometry of 270 nm equilateral side length triangle and a 50 nm tip-to-tip gap on an ITO coated glass coverslip.

**Figure 7:**
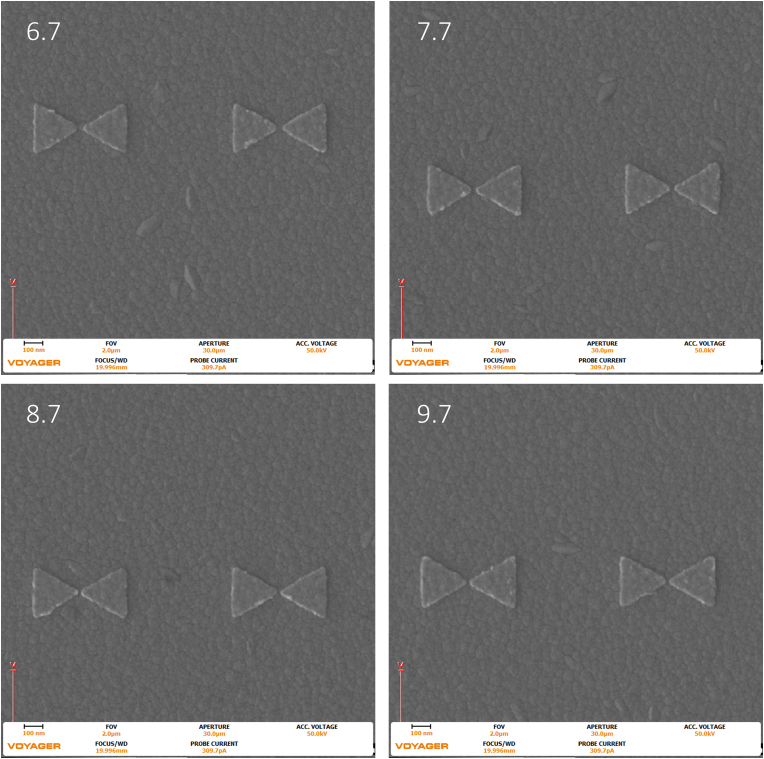
Images were taken with the Raith Voyager showing a 2 μm field of view (FOV) 6.7 (670), 7.7 (770), 8.7 (870), and 9.7 (970) Dose factor (μC/cm^2^) set of gold bowtie nanoantennas with a programmed geometry of 270 nm equilateral side length triangle and a 20 nm tip-to-tip gap on an ITO coated glass coverslip.

**Figure 8:**
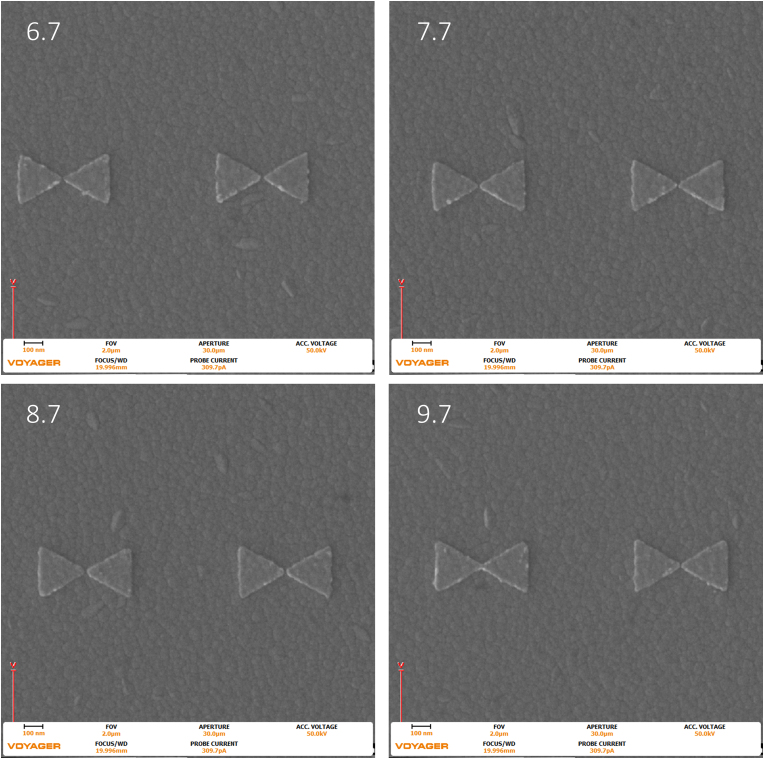
Images were taken with the Raith Voyager showing a 2 μm field of view (FOV) of 6.7 (670), 7.7 (770), 8.7 (870), and 9.7 (970) Dose factor (μC/cm^2^) set of gold bowtie nanoantennas with a programmed geometry of 270 nm equilateral side length triangle and a 10 nm tip-to-tip gap on an ITO coated glass coverslip.

**Figure 9:**
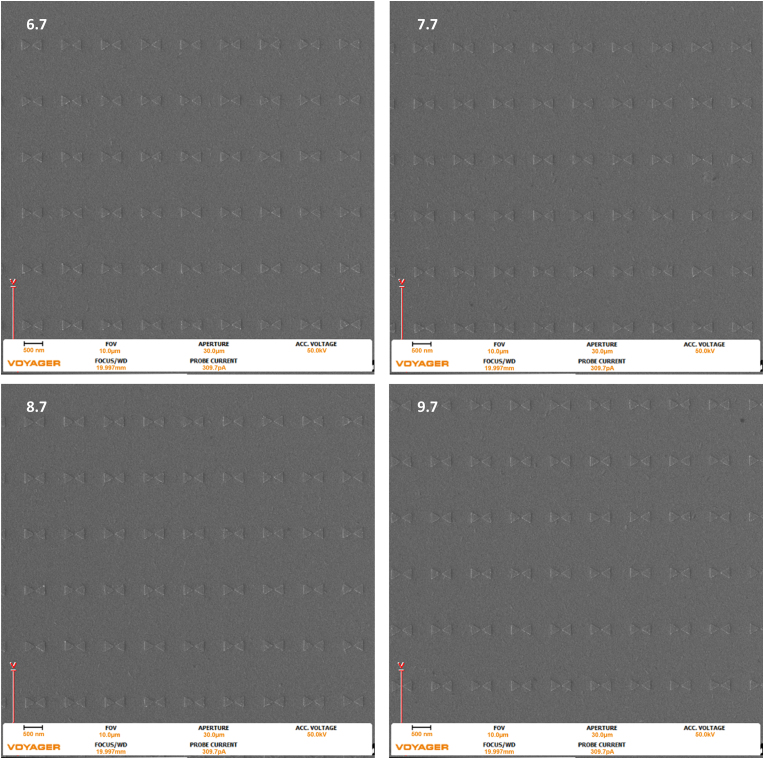
Images were taken with the Raith Voyager showing a 10 μm field of view (FOV) of 6.7 (670), 7.7 (770), 8.7 (870), and 9.7 (970) Dose factor (μC/cm^2^) set of gold bowtie nanoantennas with a programmed geometry of 270 nm equilateral side length triangle and a 50 nm tip-to-tip gap on an ITO coated glass coverslip.

**Figure 10:**
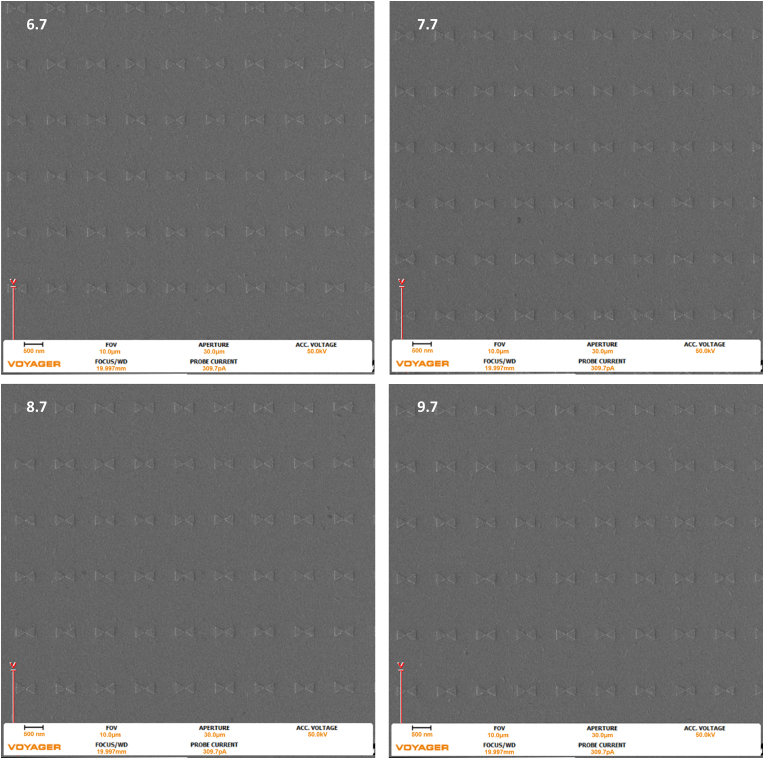
Images were taken with the Raith Voyager showing a 10 μm field of view (FOV) of 6.7 (670), 7.7 (770), 8.7 (870), and 9.7 (970) Dose factor (μC/cm^2^) set of gold bowtie nanoantennas with a programmed geometry of 270 nm equilateral side length triangle and a 20 nm tip-to-tip gap on an ITO coated glass coverslip.

**Figure 11:**
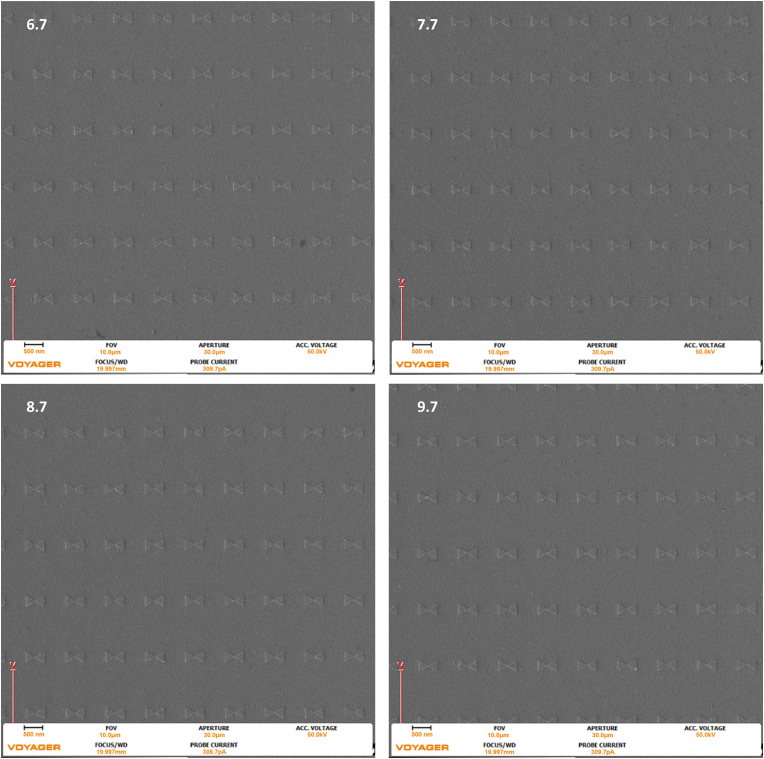
Images were taken with the Raith Voyager showing a 10 μm field of view (FOV) of 6.7 (670), 7.7 (770), 8.7 (870), and 9.7 (970) Dose factor (μC/cm^2^) set of gold bowtie nanoantennas with a programmed geometry of 270 nm equilateral side length triangle and a 10 nm tip-to-tip gap on an ITO coated glass coverslip.

From this information, it is predicted that – as the dose increases – the potential for overexposure or design related failure due to increased footprint area, such as tip merging, increases. Tip-to-tip merging is seen in the 9.7 dose factor of the 270 nm triangles with the 10 nm gap. Figure 6, Figure 7, and Figure 8 below show SEM images of the 270 nm triangles with 50 nm, 20 nm, and 10 nm gaps at 6.7, 7.7, 8.7, and 9.7 dose factors at a 2 um field of view Repeatability is understandably critical for nanostructure fabrication. Figures 9, 10, and 11, present the larger 10 um field of view (that contains Figures 6, 7, and 8 structures) of the previously described geometries. All images were taken at the center of the write field, and additionally, no additional proximity effect correction software was used during fabrication and all pattern design, programing, and pattern simulation was performed in the Raith Voyager software.

Figures 9, 10, and 11, present the larger 10 um field of view (that contains the nanoantennas seen in Figures 6, 7, and 8) of the previously described geometries. All images were taken at the center of the pattern.

The images seen above in Figure 6, Figure 7, and Figure 8 were analyzed in ImageJ, a public domain processing software for images, and the locations were randomly selected in the center of the larger dose factor field. The change in footprint area was calculated for a 10 μm field of view. The following results are tabulated below in Table 2.

**Table 2:** The Results for increasing dose with respect to the three different gaps per triangle in the 270 nm triangle bowtie nanoantennas.

Dose Factor	% increase of dose	50 nm gap %increase of area	20 nm gap %increase of area	10 nm gap %increase of area
Δ from 8.7 to 9.7	11.494	2.276	2.317	2.511
Δ from 7.7 to 8.7	12.987	0.975	2.081	2.066
Δ from 6.7 to 7.7	14.925	2.621	5.934	2.475
Δ from 6.7 to 9.7	44.776	5.979	10.644	7.218

The table above, Table 2, details the change in dose factor, the percent increase in dose factor, and the percent increases in area for each gap size. As the gap distances shrink, the percent increase in area of the footprint increases. For a 45% increase in dose, from 670 to 970 μC/cm^2^, the 50 nm gap design has a 6% increase in area, the 20 nm gap design has a 11% increase in area, and the 10 nm gap design has a 7% increase in area.

Additionally, the amount of merged structures increases as the dose increases. That data is displayed below in Table 3.

**Table 3:** The Results for percentage and amount of merged bowtie nanoantenna with respect to the three different gap sizes.

Side length (nm)	Gap distance (nm)	Dose Factor	# Structures Analyzed	# Merged BNA	% Merged
270	10	6.7	127	0	0.0
		7.7	123	19	15.4
		8.7	108	20	18.5
		9.7	108	34	31.5
	20	6.7	127	0	0.0
		7.7	113	1	0.9
		8.7	122	3	2.5
		9.7	121	8	6.6
	50	6.7	108	0	0.0
		7.7	108	0	0.0
		8.7	108	0	0.0
		9.7	131	0	0.0

The 10 nm gap nanoantenna had the most significant merged amount with 31.5% of nanoantenna tips touching across the gap for the highest dose at 970 μC/cm^2^ of 34 triangles (17 pairs) merged. This number is reduced to 18.5% and 15.4% at 870 and 770 μC/cm^2^, respectively. Notably, there were 0 merged pairs at all gap sizes for the 670 μC/cm^2^ dose. The number of merged nanoantennas has an effect on the standard deviation as seen in Table 4. The standard deviation increases with increasing dose and is approximately an order of magnitude higher in doses that had merged triangle pairs.

**Table 4:** The Results for average area and standard deviation for the three different gap sizes per triangle in the 270 nm bowtie nanoantennas.

Side length (nm)	Gap distance (nm)	Dose Factor	Average Area (nm 2)	Average Area (μm 2)	Standard Deviation (nm 2)
270	10	6.7	38336.2	0.0383	1599.7
		7.7	39285.2	0.0393	15907.1
		8.7	40096.7	0.0401	18149.0
		9.7	41103.4	0.0411	23684.2
	20	6.7	36762.3	0.0368	1584.6
		7.7	38943.7	0.0389	3975.0
		8.7	39754.1	0.0398	6567.8
		9.7	40675.4	0.0407	10727.8
	50	6.7	38143.4	0.0381	1212.5
		7.7	39143.0	0.0391	1335.6
		8.7	39524.5	0.0395	1432.9
		9.7	40424.1	0.0404	1593.8

## 4 Conclusion

This work compares the exposure doses between three different geometric spacings of bowtie nanoantennas in order to determine a dose working range in units of energy per area (μC/cm^2^). The geometries are: a 270 nm side length equilateral triangles with a 50 nm tip-to-tip gap, a 20 nm tip-to-tip gap, and a 10 nm tip-to-tip gap. The Dose factor is a multiplier applied to a constant base dose. The base dose in this work is 100 μC/cm^2^. The long molecular chains of the polymer mask undergo molecular scissioning and break apart into smaller polymer chains when energy is applied [18, 32, 33]. The nanoantennas in this work are fabricated in a five-step process: applying resist, exposure, development, deposition, and metal removal. CASINO, a Monte Carlo simulation software, was used to aid in the understanding the electron beam interactions with material boundaries and different compositions [9]. Under- or over-dosing would correspond to under or over exposure, which would then affect the volume of polymer mask dissolved. In this work are twenty separate dose arrays, ranging from 650 – 10,300 μC/cm^2^. These dose factor arrays had an internal array 7,000 bowtie nanoantenna pairs (or 14,000 standalone structures) for the 270 nm side length triangles at each the 50 nm, 20nm, and 10 nm gaps.

The appropriate dose is crucial to achieve the desired nanoscale structure. This work has shown that by increasing the dose by 44.78% there is at least a 5.9% area increase of the nanostructure but up to 10.7% increase in footprint of the nanostructure.

This experimental data supports the earlier hypothesis that by increasing the electron beam dose from 670 – 970 μC/cm^2^ there is an increase in backscattered electrons which are overexposing the PMMA mask by 5.979%, 10.644%, 7.218% for the 50 nm, 20nm, and 10 nm gaps, respectively. This overexposure causes a 15% increase in area size which could in lead to unpredictable fabrication results if performed at even higher doses. This increase in footprint can cause critical defects, like tip merging, to occur, as seen in the failed pair with the 10 nm gap. The 670 μC/cm^2^ had the lowest standard deviation and zero tip merging defects, and it is the best dose for this work, however the other doses remain serviceable. A critical defect will of the electric field generated and will generally create a failed structure. In this work a working range of 650 to 10,300 μC/cm^2^ was identified, while appreciating that the chances of a critical defect increase through the working range.

## Declarations

### Author contribution statement

Caroline Campbell: Conceived and designed the experiments; Performed the experiments; Analyzed and interpreted the data; Contributed reagents, materials, analysis tools or data; Wrote the paper.

Abigail Casey: Contributed reagents, materials, analysis tools or data; Wrote the paper.

Gregory Triplett: Analyzed and interpreted the data; Contributed reagents, materials, analysis tools or data; Wrote the paper.

### Funding statement

This research did not receive any specific grant from funding agencies in the public, commercial, or not-for-profit sectors.

### Data availability statement

Data is a part of an ongoing study.

### Declaration of interests statement

The authors declare no conflict of interest.

### Additional information

No additional information is available for this paper.
